# A bedside application-based assessment of spatial orientation and memory: approaches and lessons learned

**DOI:** 10.1007/s00415-019-09409-7

**Published:** 2019-06-25

**Authors:** Virginia L. Flanagin, Paul Fisher, Berk Olcay, Stefan Kohlbecher, Thomas Brandt

**Affiliations:** 1 German Centre for Vertigo and Balance Disorders (DSGZ), Munich, Germany; 2 Hertie, University Hospital, LMU Munich, Munich, Germany; 3 Neuro-Cognitive-Psychology, Department of Psychology, LMU, Munich, Germany; 4 Computer Aided Medical Procedures, Technical University Munich (TUM), Munich, Germany

**Keywords:** Spatial memory, Spatial orientation, Point-of-care testing, Egocentric pointing, Mobile device

## Abstract

Spatial orientation and memory deficits are an often overlooked and potentially powerful early marker for pathological cognitive decline. Pen-and-paper tests for spatial abilities often do not coincide with actual navigational performance due to differences in spatial perspective and scale. Mobile devices are becoming increasingly useful in a clinical setting, for patient monitoring, clinical decision-making, and information management. The same devices have positional information that may be useful for a scale appropriate point-of-care test for spatial ability. We created a test for spatial orientation and memory based on pointing within a single room using the sensors in mobile phone. The test consisted of a baseline pointing condition to which all other conditions were compared, a spatial memory condition with eyes-closed, and two body rotation conditions (real or mental) where spatial updating were assessed. We examined the effectiveness of the sensors from a mobile phone for measuring pointing errors in these conditions in a sample of healthy young individuals. We found that the sensors reliably produced appropriate azimuth and elevation pointing angles for all of the 15 targets presented across multiple participants and days. Within-subject variability was below 6° elevation and 10° azimuth for the control condition. The pointing error and variability increased with task difficulty and correlated with self-report tests of spatial ability. The lessons learned from the first tests are discussed as well as the outlook of this application as a scientific and clinical bedside device. Finally, the next version of the application is introduced as an open source application for further development.

## Introduction

Deficits in spatial orientation and memory are differentially related to neurological disorders and ageing. Disorientation and impaired wayfinding are often the first signs of mild cognitive impairment and Alzheimer’s disease [1, 2]. The decline in navigational ability with cognitive ageing cannot be entirely explained by a general decline in learning and memory [3]. Due to their early appearance, navigational deficits can serve as early markers for the onset of pathological cognitive decline [3]; however, they are often neglected in clinical neurology.

A unified test for spatial orientation and navigational ability has yet to be implemented into the neurological clinical routine. Many of the spatial or navigational tasks used in clinical studies involve pen-and-paper tests or virtual environments (see [4, 5] for details of the different spatial tests used). These tests cannot distinguish between deficits due to general cognitive and memory impairments, and specific deficits from sensorimotor degradation that then impact the establishment of the mental models or cognitive maps necessary for orientation in space. Clinical tests involving navigation in real environments have detected differential navigational deficits in allocentric and egocentric space [6]. However, these tests are not widely available and we just beginning to understand the systematic read-out parameters that can be extracted in real navigation paradigms [7].

Developing a standardised test for spatial ability is not a trivial task. Spatial orientation and navigation involve complex cognitive processes with a high degree of individual variability [8]. Few spatial tasks can assess the wide range of the spatial abilities necessary for successful navigation. Pointing is a commonly used tool for measuring environmental spatial abilities, i.e., the ability to update one’s position in large-scale or environmental space [9]. It can be used to measure spatial deficits in nearer space (vista space) as well as far space (environmental space) [10, 11]. It can measure differences in performance between the horizontal and vertical planes [12] or hemispheric differences that can be present in specific patient populations [13]. A pointing task can be varied to test many different aspects of spatial ability (i.e., spatial cues, computational mechanisms, and spatial representations within mental maps) [8]. For instance, the processing and memory of spatial environmental cues can be tested simply by pointing to previously viewed objects in the room and pointing after imagined shifts in perspective can address spatial updating. Importantly, pointing is one of the few spatial tasks that correlates well with the self-reported measures of spatial ability [9]. For these reasons, pointing was chosen as the most suitable task for a bedside assessment of spatial ability.

The mobile phone is a widely available device with the potential to test spatial ability, but their use in navigation research up until now has been scarce. Recently, however, a video game application assessed navigational ability in a virtual environment on a mobile device and thereby collected the largest and most diverse cultural navigational data set that exists to date [14]. In addition to navigating through a virtual environment, mobile devices can measure spatial information through a combination of electronic components, including the global positioning system (GPS), gyroscope, accelerometer, and compass components (magnetometer). These components provide variable spatial information such as the angle of the devices position with respect to north, the trajectory the device has taken, and the position of the device in the world. Health care professionals are increasingly using mobile devices in the workplace, for medical data collection, storage, and for point-of-care testing (POCT) [15]. In summary, the pervasive presence of mobile phones in a clinical setting, together with their ability to measure spatial information, makes them a reasonable device option for a bedside test of spatial ability.

**Fig. 1:**
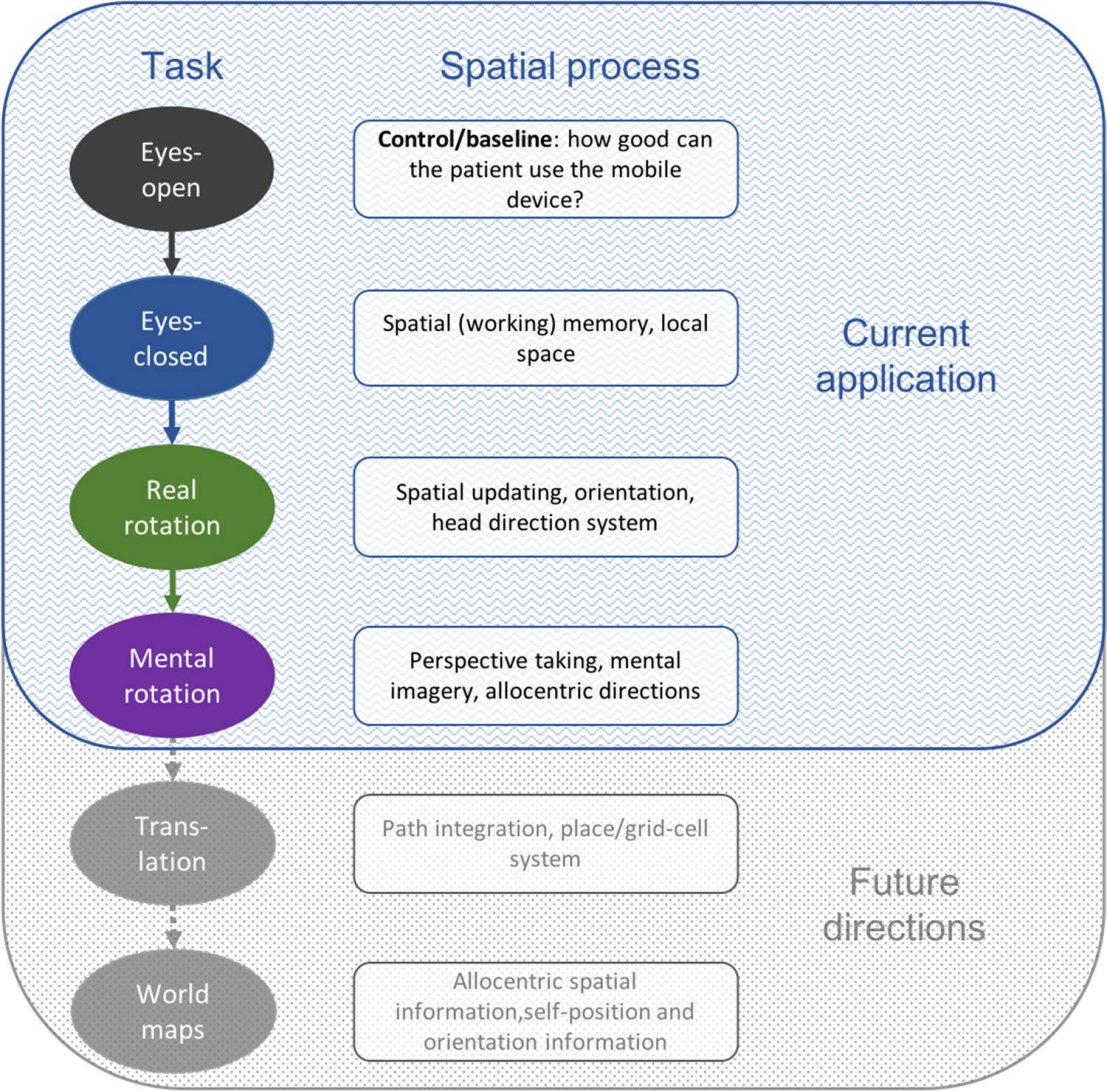
Current and future goals of the spatial bedside tool. The goal of the application is to test different aspects of spatial processing in virtually any setting using a mobile device (in this case an iPhone). In the current application, differences in degrees, pointing angle, and variability of pointing are tested against the baseline condition (eyes-open—black). Base spatial memory is tested with eyes-closed (blue), and spatial updating (green) and perspective taking (purple) are tested with real and mental rotations. In future versions of the application (in gray here), we plan to add pointing after translational movements and pointing to well-known regional landmarks (e.g., the center of the nearest city)

The goal of the current study was to test the feasibility of a test for spatial orientation and memory using pointing with a mobile phone. Our approach was based on our previous experience pointing towards invisible targets in a multi-level building [12]. Figure 1 shows the breakdown of individual tasks that were envisioned and the associated spatial process which they represent. The pointing tasks that are the most feasible in a bedside setting are shown at the top in blue. Each ellipse represents an experimental condition that can be tested with pointing. The level of difficulty of each condition increases from top to bottom, each reflecting an additional spatial computation used in navigation. A memory component is present in all test conditions (not the baseline) that consists of a sensory-motor command and a memory of the location of targets in space. At the bottom in grey are additional potential parts of the test, such as translations, that will be important to test for in patient populations [8] but that were not examined here.

A mobile application (app) was programmed to access the information from the sensors currently built into most mobile phones in real time. With this application, the variability and reproducibility of pointing responses was tested in a cohort of healthy young controls. Reproducibility was tested by collecting data from the same participant on multiple days. Then, pointing error and pointing variability were compared between individuals’ targets and conditions. Individual pointing performance was then compared to documented neuropsychological tests for spatial ability. Based on both the data collected and in-depth post-experiment interviews with the participants, we identified the current limitations and future directions for the mobile application.

## Methods

### Participants

Twenty healthy adults (age 20–30, 14 females) took part in the study. Participants were recruited via word-of-mouth and email from the LMU Munich and the German Center for Vertigo and Balance Disorders (DSGZ) at the University Hospital Munich. Thirteen participants were right-handed, and 15 were right-eye dominant (“Procedure” for how this was assessed). All participants had between 5 and 10 years of mobile phone usage. Participants gave written informed consent in accordance with the Declaration of Helsinki and were monetarily compensated for their time at a rate of 10 Euro/hr. Upon inclusion in the study, the participant was assigned to rotate (real rotation) either to the right or to the left.

## Experimental setup

**Fig. 2:**
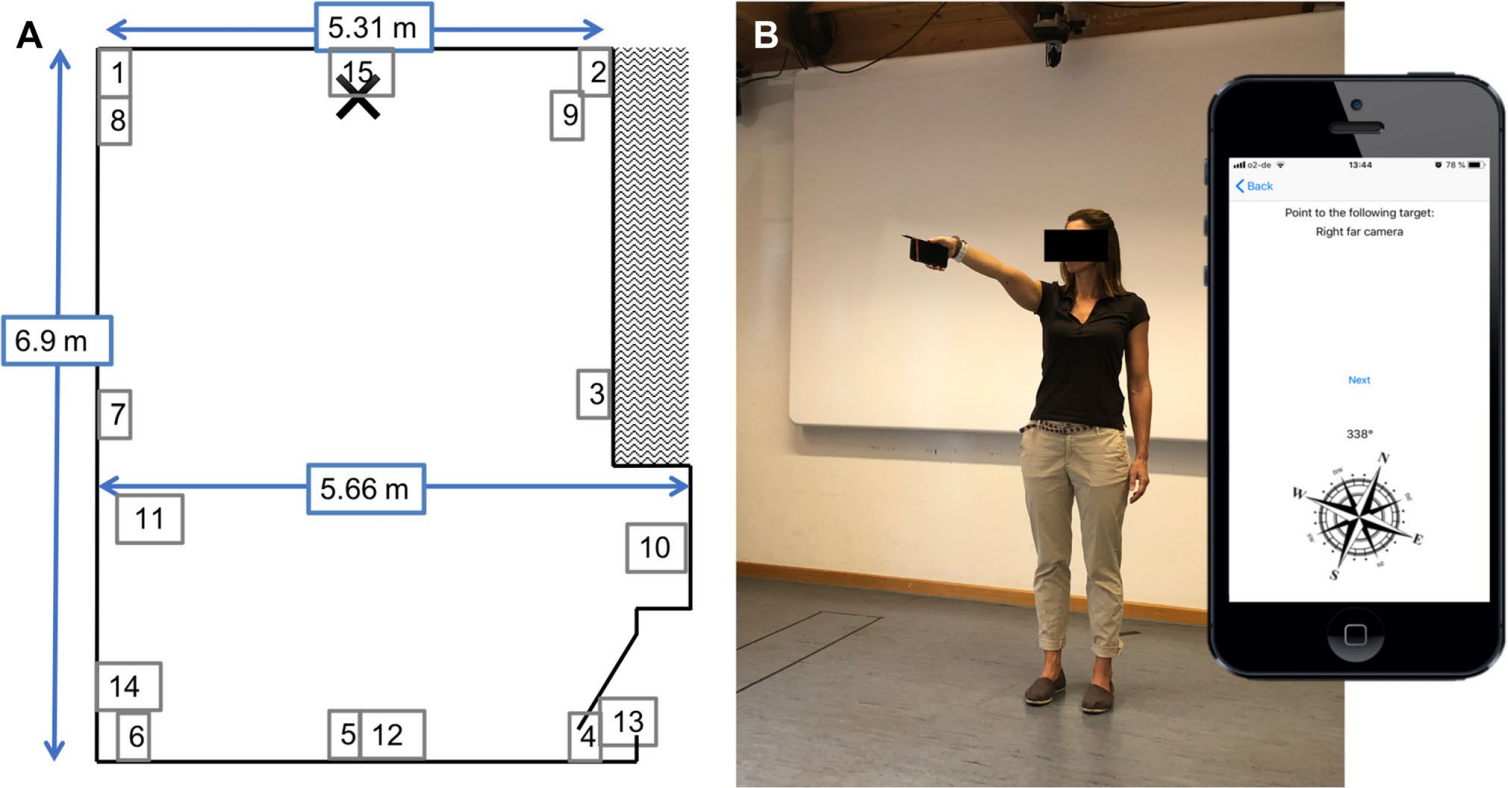
The experimental setup and pointing app. **a** An example participant pointing to a frontal right target (target 6 in **b**). Inlaid is a picture of the app as seen by the participant. **a** target is shown, as well as a compass and the current azimuth position. **b** The layout of the room used for testing and the horizontal target positions. Participants stood at the blue cross and pointed to the various targets. The dimensions of the room are given in meters. Target numbers correspond to the names of the targets in Fig. 5. Images of example targets can be seen in Figs. 3 and 4

The tool used for this experiment was a self-written application for iOS (version 10.3.3) that was installed on an iPhone 5S. The application referred to as the “pointing app” was developed by SK. The application was created in Swift (Version 3) in X-Code Version 3.2, for MacOS versions up to 10.12. The target names (see Fig. 5 for names) and presentation order were hard coded into the application. The application included a graphical user interface for entering patient information including the patient’s gender and birthdate. A white screen appeared for each target with the name of the target at the top of the screen as well as a compass with the direction away from north in degrees above it (Fig. 2a—inlay). Once the application was started, the heading of the phone as measured by the compass application of the phone was continually measured and updated until the participant pressed the middle of the screen to confirm their pointing direction. The compass information was converted into a three-dimensional (3D) unit vector representation of the last pointing direction at the end of a trial. This value was then saved to memory along with the name of the target position. A JSON file with all of the data in the order collected was sent from the phone to a personal computer to be analysed offline.

A medium-sized room of approximately 6 × 7 m was used throughout the experimentation (Fig. 2a). Fifteen targets were predefined as well as the participant’s location at the far end of the room (Fig. 2a). The targets were chosen to be at different heights and different horizontal locations with respect to the participant. The targets covered approximately 180 degrees azimuth from 90 degrees to the left and the right of the participant and approximately 110 degrees elevation from approximately 20 below straight ahead to straight above the participant. Targets were presented in a predefined and pseudorandomized order for all conditions, where right and left targets were presented in alternating order. A pencil was affixed to the side of the mobile device to aide in accuracy of pointing (Fig. 2b).

Four experimental conditions were used (Fig. 1). In the first condition eyes-open (EO), participants kept their eyes open throughout testing and read the targets one from the screen before pointing to target and then moving to the next target. It is not feasible with current mobile phone technology to determine the “ground truth” about the desired target location within a building. Therefore, the EO condition, i.e., pointing to visible objects within a room, served as a baseline for all other measurements. The second condition was eyes-closed (EC), in which participants remained where they were and pointed to the same targets as in EO. In all conditions where participants kept their eyes-closed, the experimenter gave participants each target name verbally. The third condition was real rotation (RR), where participants rotated to the right or to the left (counterbalanced across participants, gender, and handedness) 90 degrees and then pointed to the same targets. Their eyes remained closed throughout this condition. Rotations of 90-degree angles were used to reduce bias, as individuals to bias angle estimates toward 90 degrees [16]. Mental rotation (MR) was the last condition measured. In this condition, participants remained facing the same direction as for EO and EC, but imagined being rotated to the right or the left 90 degrees. If a participant performed RR to the left, they performed MR to the right.

### Procedure

The experiment was completed by each participant on two consecutive days. After agreeing to participate in the study but before beginning the pointing experiment, participants completed all of the self-report questionnaires, except the Wayfinding Strategy and Spatial Anxiety scale. A variation of the Miles test [17] was used to assess the dominant eye. Participants formed a small triangle with both hands with their arms stretched out as far as possible and asked to focus on a mark on the wall completely within the boundaries of the triangle. By alternately closing one eye and then the other, the dominant eye was determined as the eye through which they could still completely see the object.

After these tests, participants read through a detailed written instruction sheet for the experiment. The important points were then reiterated by the experimenter. Participants were given freedom to choose which hand they used for the pointing application. Only two participants chose to use their left hand, all others used their right. On the first day, participants performed eight repetitions of all 15 targets with eyes-open (EO) (120 trials). Two repetitions constituted starting the iPhone application once. During testing, each target was presented on the screen and participants pointed to the target. Once the participant was happy with the pointing direction, they pressed the screen to save the pointing direction. The next target was then immediately presented on the screen. After two repetitions, the app presented the data on the screen which were saved as JSON files to the iPhone and then emailed to a personal computer. After two repetitions, the participant was given a short break ( ~ 1 min) if desired. Once a condition was finished, participants were given a break of approximately 5 min before starting the next condition. All repetitions of one condition were completed before continuing to the next condition. After EO on day 1, participants were asked to perform six repetitions of the same targets but with their eyes-closed (EC condition) (90 trials). As before, a short break was given after each pair of repetitions. Whenever the participants had their eyes-closed for the task, the targets were read out by the experimenter.

On the second day of the experiment, six repetitions of each target were performed for three conditions in the same manner as the first day. First, participants performed the EO condition (90 trials), to look for test–retest reliability. Then, both real and mental rotation (RR and MR) conditions were performed, in that order, with closed eyes. For the real rotation condition, participants rotated 90 degrees either to the right or to the left (this was determined at the time of inclusion to balance for right and left turns across participant, gender, and handedness). They then performed eight repetitions of pointing to each target from the rotated position. For the mental rotation condition, participants were facing forward but imagined being turned to the opposite direction as their real rotation condition. They then performed the eight repetitions again with closed eyes, as if they were rotated 90 degrees.

After all of the pointing tasks were done, participants completed the Wayfinding Strategy and Spatial Anxiety scale. Outcome values of these tests correlate better with experimental performance when they are given after the experiment [18]. Finally, participants were verbally asked about their experience using the mobile application and probed whether they could imagine anything to improve usability.

## Self-assessment questionnaires

To compare the relative pointing performance to other measures of spatial ability, a number of self-report spatial perception and navigation questionnaires were also administered. These included the Santa Barbara Sense-of Direction scale (SBSOD) [9], the Perspective Taking test [19, 20], a modified version of the Wayfinding Strategy scale, and the Spatial Anxiety scale [21]. The SBSOD scale provides a self-report measure of an individual’s navigational ability, by asking participants to judge 15 statements such as “*I am very good at judging distances*” as to how much they agree with them on a seven-point Likert scale [9]. In the Perspective Taking test, participants are asked to draw a line describing the pointing angle from a current position and orientation to a third object. Participants have 5 min to complete as many as possible of 12 situations and a final score is composed of the pointing errors [19].

In the post-experimental debriefing, participants were asked multiple questions about the usability of the app. First, they were asked whether they could remember the order of the pointing targets and if so how many, to see if the fixed randomisation may have improved participants’ performance. Participants were asked about the difficulty of individual targets, and whether lateral/frontal or left/right targets were more difficult to point to. The difficulty of conditions was then asked and why, and finally, they were asked whether they had any suggestions for improvement of the test.

### Data analysis

**Fig. 3:**
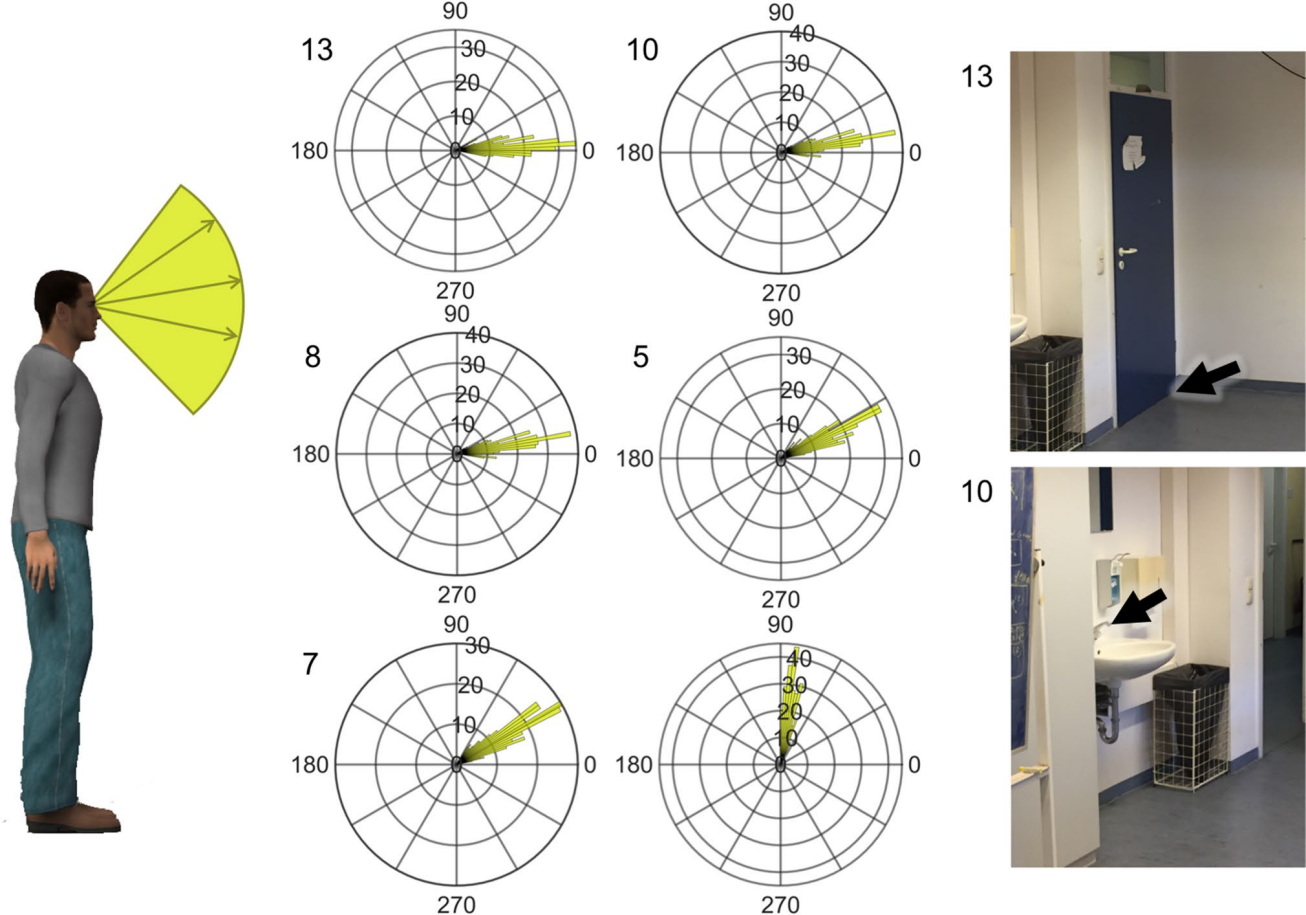
Vertical (elevation) pointing angles for six example targets during eyes-open (EOC). The mobile pointing application reproduces similar pointing angles across participants for each target. Left: a schematic of what the angles on the polar histograms represent. 90 degrees would be straight up and 0 is straight ahead. Middle: the polar histograms without averaging for all trials and all participants for six example targets that cover the entire vertical range of targets. Right: images of two example targets with numbers matching the numbers on the histograms and in Figs. 2, 4, and 5. Black arrows show the target participants were asked to point to

Data were analysed using Matlab (R2018b: 9.5.0.944444). The output of the pointing app for each target for each trial was the three-dimensional polar vector of the pointing angle on a unit sphere, given in values between -1 and 1 saved in a JSON file. These three values represent the direction of a unique vector or the pointing angle. The coordinates corresponded to East, North, and Up, respectively (See Fig. 3). Data were extracted from the JSON files using the JSONlab toolbox for Matlab. The 3D pointing vector was converted to pointing angles in azimuth (horizontal plane) and elevation (vertical plane). Mean pointing angles and standard deviations of pointing angles were calculated using circular statistics using the circstat toolbox for Matlab [22]. Cronbach’s Alpha was tested using the intraclass correlation coefficient function (ICC.m) as defined in McGraw & Wong [23]. For pointing errors compared to the baseline condition, the circular distance was calculated between the average baseline pointing direction and the angle in each trial individually. For each participant, condition. and target, the pointing directions or error was tested for uniformity and compared to a von Mieses distribution for normality before testing. Statistical tests using pointing angles or errors were calculated using circular statistics; non-circular statistical tests were used for the standard errors. Pearson’s correlation coefficient was calculated when comparing the result of the self-report tests to the performance on the pointing app.

## Results

### Reliability and pointing measures

All of the participants completed all of the tests on both testing days. The reliability of the eyes-open or baseline condition was examined across days and participants for all targets. The internal reliability, as measured with Cronbach’s alpha, was very high across the 15 target locations for the eye-open condition (EOC) for horizontal pointing angle (*α* = 0.9936 CI = 0.9878–0.9975) and vertical pointing angle (*α* = 0.9978, CI = 0.9957–0.9991). The test–retest reliability in the EOC between days 1 and 2, and found that it was also high (*α* = 0.9652 CI = 0.9622–0.9669).

**Fig. 4:**
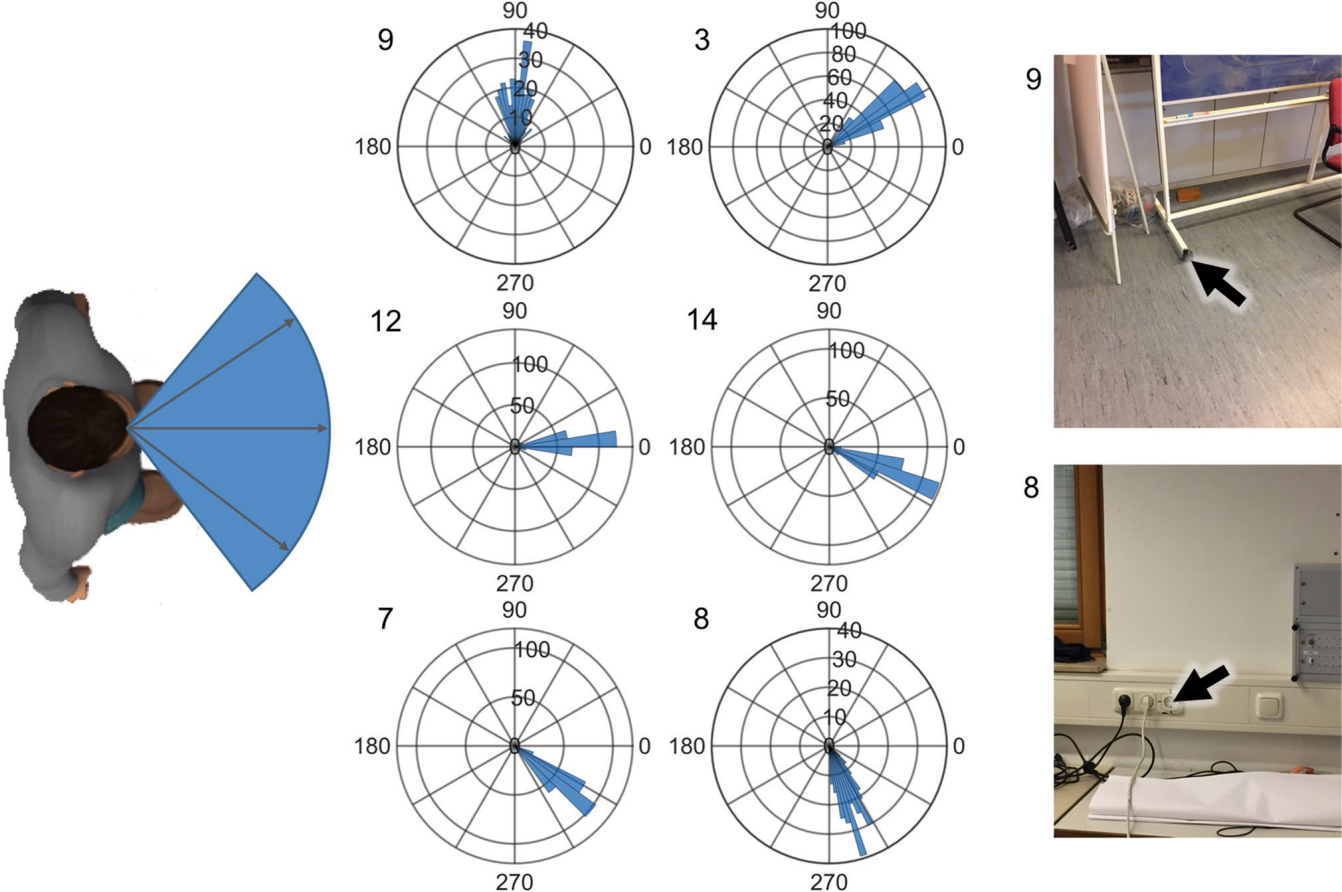
Horizontal (azimuth) pointing angles for six example targets during eyes-open (EOC). Description as in Fig. 3. Here, − 90 corresponds to left 90° from straight ahead and 90° correspond to 90° to the right. Numbers correspond to targets in Figs. 2, 3, and 5

Although we did not measure the physical location of the targets themselves and, therefore, have no ground truth about the actual pointing vector, individual targets could be easily distinguished from one another and are consistent across participants and days. To graphically demonstrate this, we plotted the distribution of pointing angles during EOC for all participants and all repetitions as 2D vectors in elevation (Fig. 3) and azimuth (Fig. 4) for six of the fifteen targets tested. The targets are ordered from left to right and lowest to highest, respectively. As can be seen in the polar histograms, the pointing angle is consistent for each target, as most of the angles fall into few bins. All of the targets showed a distribution of pointing angles that were significantly different from a uniform distribution, suggesting that the measurements from the device were random, but towards a specific direction.

**Fig. 5:**
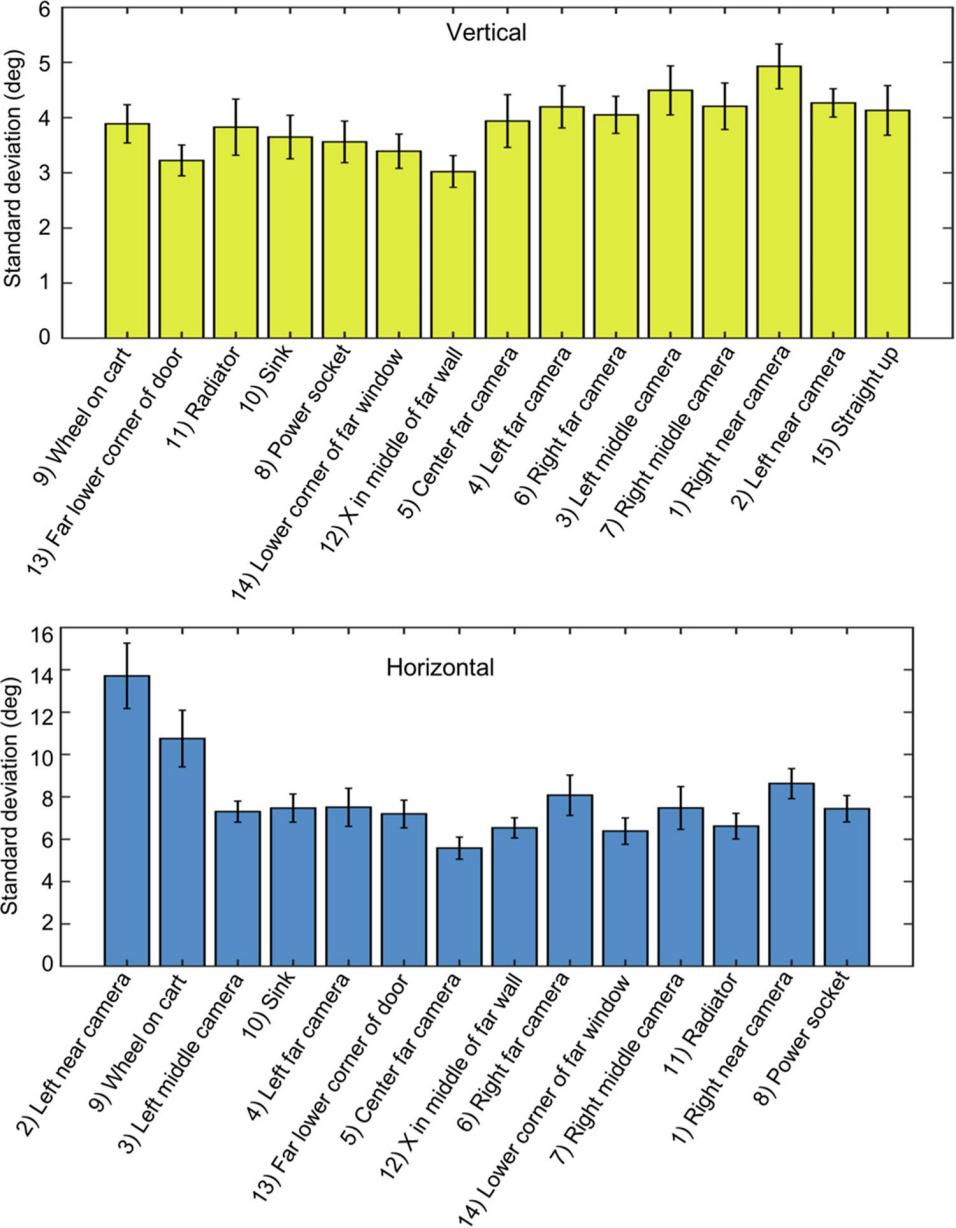
Average variability of pointing for each target during eyes-open. The standard deviation of pointing direction in elevation (top) and azimuth (bottom) for each target for each subject. For each of the figures, the targets are arranged from lowest to highest and from left to right, respectively. Vertical bars represent average ± standard errors. Variability was, on average, lower in elevation than in azimuth. Targets to the side opposite the pointing hand showed higher variability in azimuth. The target straight up was only analyzed in elevation, as any variability in azimuth is inconsequential

The variability in pointing between repetitions of the same target within each person for EOC was measured by the standard deviation (SD) of pointing angles. An application that tests spatial ability should, if possible, have low within-subject variability for each target separately. We found that the average SD of pointing across all targets was 3.83 ± 0.49 (SD) deg. for elevation and 7.93 ± 2.13 (SD) deg. for azimuth. Figure 5 shows the SD, averaged across participants, for each of the targets, for elevation (top) and azimuth (bottom). The variability in vertical pointing did not show any systematic effects related to the location of the targets. Pointing variability in the horizontal showed a significant increase in pointing variability to the left side of the room, for the two most eccentric pointing directions, that are approximately at 90 deg. to the left of straight ahead. This increase in variability is likely due to the fact that most participants (all except for two, see “Procedure”) used their right hand to hold the mobile device for pointing, a previously described phenomenon [24].

## Test conditions

**Fig. 6:**
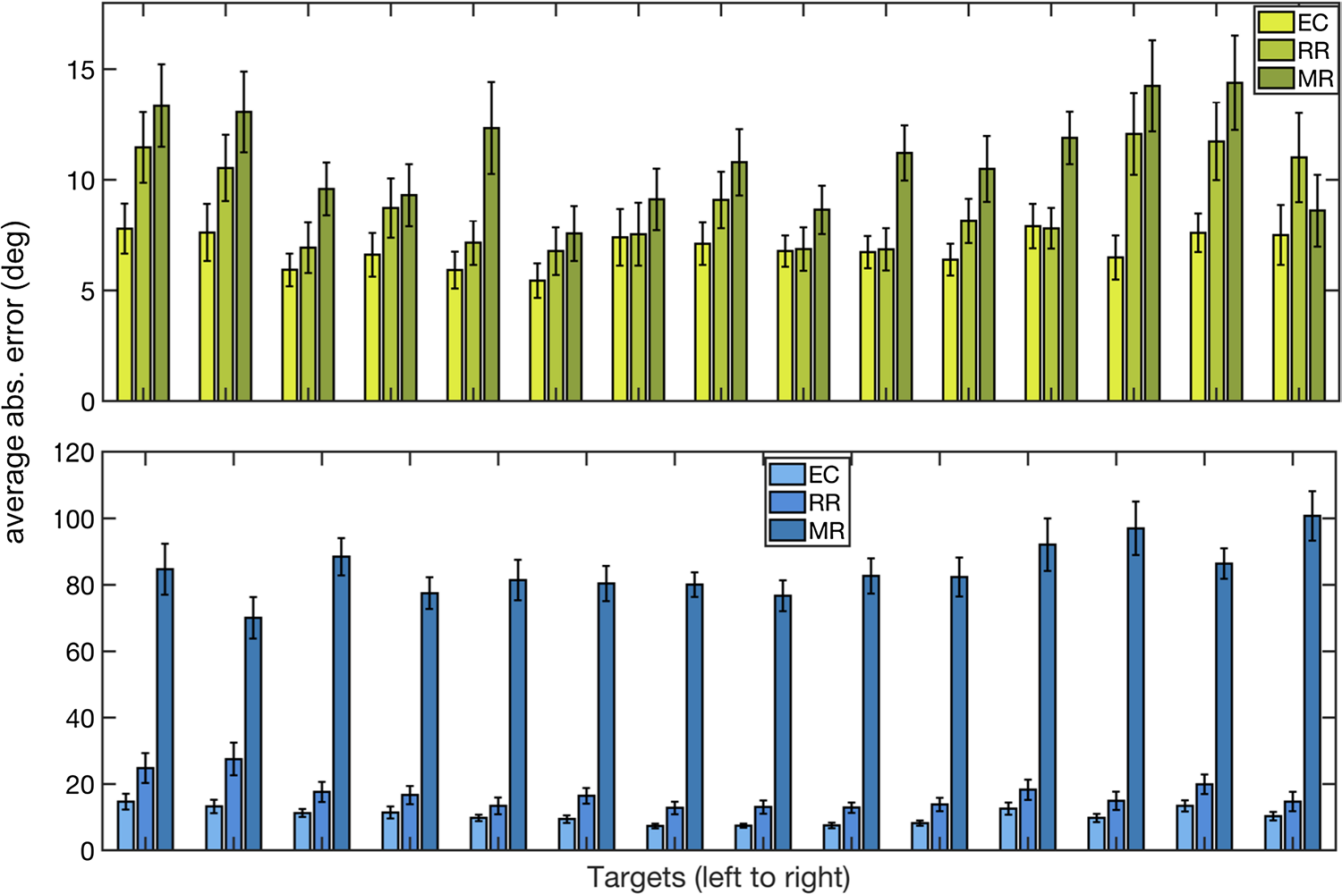
Pointing error for the three test conditions. The standard deviation of the three test conditions was compared to the same target in the eyes-open condition. The variability of pointing increases dramatically from eyes-closed to physical rotation and then again in the mental rotation condition. Variability is measured by the standard deviation of pointing across trial of the same target. Mean and standard error bars across subjects are shown. *EC *eyes closed, *RR * real body rotation, *MR *mental body rotation

Pointing error and variability for the test conditions, eyes-closed (EC), real rotation (RR), and mental rotation (MR) were then examined. To look at the errors in pointing, the direction of the pointing vector for each target in each trial was subtracted from the average pointing direction in the EOC. The resulting average error for each target can be seen in Fig. 6. The vertical pointing error was much smaller across all conditions than the horizontal pointing error (Fig. 6). A one-way rm-ANOVA showed a significant effect of condition [F(14) = 3.27, *p* = 0.04] for horizontal pointing error, with an increase in pointing error with increasing difficulty. A similar but less pronounced effect was seen in the vertical pointing errors. The pointing errors were very high for MR, which is likely due to 90 or 180 degree pointing errors that occurred in most participants in all targets. Indeed, the data in the MR condition showed a bimodal spread, meaning that the mean pointing error may not be a useful measure or pointing error in MR, and it may be more informative to model the data and extract moments from the model fits.

Similar to the pointing error, the variability of pointing increased across each condition as the conditions became more difficult, such that MR had the largest standard deviation. Fortunately, no systematic effect of turning direction (left or right turns) could be seen in either of the rotation conditions, suggesting that right and left turns can be considered equally.

## Relationship to existing spatial tests

We then looked at whether the performance on the pointing task was correlated to the pen-and-paper questionnaires or tests that the participants performed. No correlation was found between the variability in pointing in the baseline condition EOC and any of the pen-and-paper tests tested. The baseline condition should not be affected by individual variability in spatial orientation performance. However, we did find a negative correlation of the pointing error in EC and the self-report questionnaire for spatial navigation (SBSODS), suggesting that the EC condition already assesses spatial orientation ability. We also found a weak correlation between the performance in rotation and the perspective taking test, suggesting that the egocentric rotational performance was partially related to object-centred perspective taking, which has been seen in the other pointing tasks [19]. Together, it appears that the inter-subject variability seen in the output of the pointing app is related to individual variability of spatial performance, and sensitive to different aspects of pointing, although the underlying abilities may still be different [19, 20]. Because we did not counterbalance for gender when selecting our participants, we did not look for gender effects here.

## Feedback

All of the participants were debriefed after the experimentation was over and asked for feedback on their experience with the App. All participants reported that the mental rotation condition was the most difficult of all of the conditions in the pointing task. No consensus about the difficulty of the location of individual targets was found, with regards to near and far, or right and left. Most participants noticed that the order of targets was always the same; however, except for the first and last targets and for some participants the 1–2 targets after straight up, no participants remembered the order of all of the targets. An important feedback from the participants was about the verbal commands for all of the test conditions. Many participants commented that the voice of the experimenter provided an auditory landmark cue that they used for grounding their mental model of the space, especially for the more difficult rotation conditions. The experimenter consequently moved between targets for each subject; however, future versions of the application should include verbal commands from the pointing application itself.

## Discussion

In this study, we examined the feasibility of using pointing, measured on a mobile device, for testing spatial ability in a bedside setting. First, we established that the eyes-open condition, which served as a baseline for all other test conditions, showed a high reliability and reproducibility across days, and targets were well separated. The only weakness was that the pointing in the horizontal plane was more variable than expected. Variability in the baseline condition was half as large, approximately 4 degrees SD, in the vertical plane compared to the horizontal plane, although range and sampling effects may partially account for the differences found [25]. Strongly eccentric targets (around 90°) contralateral to the pointing hand had a higher variability and a larger pointing error in the horizontal plane than ipsilateral and frontal targets. If these targets are left out, the difference in variability between horizontal and vertical pointing was only 1.5. In the test conditions, pointing error and variability increased with the difficulty of the task, from eyes-closed to real and then mental rotations, as expected from our design (Fig. 1). Pointing errors in the vertical plane remained low, with errors around six degrees for eyes-closed, and up to 14 degrees for mental rotation for young healthy adults. Mental rotations were by far the most difficult in the current application: the errors were the highest, and in the post-experimental debriefing, all participants reported mental rotation as the most difficult condition. In summary, with a few crucial modifications, the mobile pointing application provides a useful tool to bring spatial testing to the clinical routine.

Because of the myriad spatial deficits in the early stages of multiple neurological disorders and normal cognitive ageing, a fast, standardised test for spatial ability would be a desirable addition to diagnostic testing [13]. The underlying brain structures and cell types involved in various types of spatial ability are known and show deficits in cognitive ageing as well as in pathological disorders [5, 26, 27], which, together with a test for spatial ability, may help us to better understand the disease progression and the nature of the cognitive deficit. Already in the first condition of the current application, individuals must develop a mental model of the environment that they are in, and then use that model to accurately point to remembered targets in space.

Pointing was chosen for this tool as it is a widely used task to assess large-scale spatial ability that can be used to systematically test multiple aspects of spatial cognition [9, 10]. The outcome measures collected through pointing are more related to the underlying spatial skills than many other tests for spatial ability [28]. Pen-and-paper tests, such as the Mental Rotation Test and the Arrow Span Test, do not often correlate to actual navigational ability, because they operate in peripersonal space, i.e., the space in which we can grasp objects, which is processed differently than extrapersonal space [28]. Although the pointing application is more closely related to real navigation performance, in its current form, it is still limited to pointing in vista space. All of the targets tested were visible to the participant, when their eyes were open, from the same vantage point. Unfortunately, the performance in vista space is not entirely transferrable to behaviour in environmental space, the space that is pertinent to large-scale real navigation [29]. However, the advantage of application can easily be adapted to test large spatial scales by testing pointing to landmarks in the external environment, such as the entrance to the clinic, the center of town, or famous landmarks (see the future directions in Fig. 1).

The results of the current application support the previous evidence of an anisotropy between vertical and horizontal spatial orientation abilities [12, 30]. Contrary to the previous studies, the vertical pointing error in the current study was less than horizontal pointing error. The previous studies have shown significantly worse performance for vertical spatial navigation [12, 30, 31]. The difference between the current results and the previous results is likely because the current study was the only study to examine performance in vista space, and all other studies were conducted in environmental space. Most rooms are wider or longer than they are tall. The range of possible vertical target positions in a room is limited to slightly more than 90 degrees, whereas horizontal pointing targets can range more than 180 degrees. Range effects [25] are a likely cause of the differences between vertical and horizontal pointing found in the current study. Whether the differences in performance are related to different neural substrates as in [30] will be seen through testing of patients with varying lesions.

Many studies differentiate between egocentric (self) and allocentric (other) spatial representations through different tasks and associate these types of navigation with a specific brain region [7, 32, 33]. Specifically, the medial temporal lobe (MTL), including the hippocampus, is associated with allocentric navigation and the concept of a cognitive map [34]. Most of these spatial tasks, however, can actually be performed using a combination of allocentric and egocentric representations or coding schemes, and a growing body of evidence contradicts a strict dissociation between allocentric and egocentric behaviour and neural mechanisms [11, 35]. In the Morris Water Maze (MWM), for instance, although classically considered allocentric spatial navigation that is disrupted in animals with hippocampal lesions [36], it is possible to solve the task based entirely on visuo-motor strategies and viewpoint matching without a clear ego- or allocentric representation [11]. In the current study, the pointing tasks were clearly in an egocentric reference frame, i.e., with respect to the navigator’s axis of orientation [37], but, in all of the test conditions, participants can use relative distances and orientations between targets, i.e., an allocentric reference frame, when recalling target locations. A combination of egocentric and allocentric reference frames is the most likely in experimental conditions in which spatial updating occurred, i.e., with mental or real rotations. Based on the previous work [27], we believe that these conditions, and the future translational condition (Fig. 1), although not specifically allocentric in nature, will be able to illuminate spatial deficits based in the MTL. In addition, if clinicians wish to test in an allocentric reference frame, participants could produce angles from one target to another, as in the Perspective Taking Task [19] using the same mobile application developed here.

Virtual reality (VR) is an alternative to pointing that has found increasing usefulness in studying spatial navigation across many different groups of people. A VR experiment programmed as an application for mobile phones made it possible to measure from a very large cohort of individuals across the entire lifespan and much of the globe [14]. However, VR can be problematic for testing spatial abilities in patient populations. Older adults have less experience with VR and the resulting stress and anxiety when using VR may not be desirable if the individual is in a patient situation [38]. Navigational performance generally decreases in VR, although trends across age and disorder are often maintained [5, 39]. Accuracy is generally higher in the real world, and VR environments typically have a mismatch information in one or more sensory modalities [40, 41]. Therefore, differences in performance in VR between patient and control populations can be difficult to interpret, especially for sensorimotor and vestibular disorders [40, 42]. A higher level of immersion, including 3D visualisation and auditory or proprioceptive input, appears to reduce cybersickness, which is more common in older adults [5]; however, complex VR environments are typically not feasible in a bedside setting.

A mobile pointing application for clinical spatial orientation testing provides a complimentary method when VR environments are not practical. Mobile phones are already being tested to detect falls or determine physical activity [43]. The use of mobile applications in medicine has become highly pervasive; over 80% of medical practitioners in USA use some sort of smartphone or tablet in their workplace [15]. Although mobile applications for medical use, termed Mobile Health Applications, mostly focus on fitness and self-monitoring [44], mobile applications specifically for health care professionals can improve information management and clinical decision-making [15]. This test for spatial ability takes advantage of the ubiquitous presence of mobile devices in a health care setting. Unfortunately, the evaluation of mobile health applications such as the CRAAP (currency, relevance, authority, accuracy, and purpose) test or ethical considerations have focussed on user-/patient-based mobile applications [45, 46], and is less useful for evaluating the current application.

## Lessons learned

A number of lessons can be taken away from these initial results. First, the choice of target is important. Targets with clearly visible but small target areas are preferable as they reduce variability in the baseline condition. For instance, the corner of two walls and the floor is a well-defined target, whereas the large objective of a camera that is 10 cm diameter is less well defined. Targets that are more eccentric than 60 degrees from the midline will be more error prone and more variable and should, therefore, not be used.

Second, the target order should be randomised. In the initial version of the application, randomisation was not possible. Over the testing period, the participants learned the order of the targets, although not entirely. Motor sequences of 20 spatial targets can be learned after only 25 trials, and that this learning lasted multiple days [47]. Randomising the order of targets will disrupt this type of sequential learning.

Third, although the pointing variability was low in our sample of young adults, further reductions in variability would be desirable. One way to improve accuracy would be to add additional visual information to the baseline condition. Pointing is a highly complex motor process, involving an internal model of the end-to-end pointing vector that differs between individuals [10, 41, 48, 49]. Pointing with a mobile device is not a trivial extension of pointing with a finger. In a small set of pilot participants, we found that a laser pointer affixed to the mobile device could further decrease pointing error.

Fourth, for all of the eyes-closed conditions, the application should provide auditory commands. The experimenter’s vocalisations, which always came from the same location, provided an auditory landmark that aided in pointing in the test conditions.

Fifth, the use of additional sensors will improve the accuracy of the device itself. The current study only used compass information to calculate a pointing vector. During testing, we discovered that a 180-degree flip occurred in strongly downward directions. This was also present when using the manufacturer’s compass application and was not improved by recalibrating the compass. This issue may be specific to the iPhone 5 s and we are currently testing the application on different mobile phones. Until then, targets close to the patient on the floor should be avoided.

## Outlook

Based on the lessons that we learned from this first pilot study, we have created a modified version of the pointing application for iOS. This is called the Pointing App and is available, with this data set at https://github.com/BerkOlcay/PointingApp. The following improvements have been made:

1. Targets and commands can be presented verbally from the mobile device itself, in either English or German.
2. The test person can use the volume buttons on the side of the device to choose the pointing location.
3. The graphical interface is more user friendly. Targets can be added to the application via the interface, which are saved for future testing. The number of repetitions and the condition tested are selected from a drop-down menu and part of saved data.
4. The output files can be saved as text files or as JSON files either in alphabetical order of the targets (for diagnosis), so that repetitions of the same target are together, or in the order that the targets were presented (for research).
5. GPS information is also saved with each target. The GPS signal is only accurate to 5–8 m [50], making it too inaccurate for distance measurements. However, the GPS position can be used to calculate pointing angles to well-known targets outside of the building (i.e., a relevant landmark in a city, the next subway station) or global landmarks, providing an expansion to the current study.

First, in pilot testing on patients, we found that the transition time between targets was too fast and we are currently adding a delay between targets. In addition, the data and analysis pipeline have been made available at https://web.gin.g-node.org/. Future versions of the application will integrate the analysis into the app, so that medical professionals will have a direct read-out of the data immediately after the test is administered.

The current study only tested healthy young individuals. The functionality of the test has yet to be examined in patient populations. However, we can recommend the following modifications to the current procedure for patient populations.

1. Perform all of the conditions on 1 day.
2. Use only five or six targets, including targets that are straight ahead and up, and targets that are to the right and left of the patient, but not more than 60 degrees lateral.
3. Five-to-six repetitions for each target and condition will likely suffice for a clinical test. Together with a reduced number of targets, the test should not take more than 30 min, making the test more feasible in patient populations.
4. Start with the easiest condition and work up to the most difficult condition. If, at any point, it becomes clear that the patient cannot complete the condition, then stop the test there.

Using this modified software, we have begun testing usability in patient populations. With the application in its current form, we will be able to examine differential effects of translational and rotational movements. The first step towards this goal involves collecting data from a large cohort across all age groups to differentiate between pathological deficits in spatial ability and normal ageing [27]. Defining normative values for the different conditions across the entire adult lifespan is crucial for the clinically applicability of the test. By making this application open source, we hope to bring data together from multiple institutions to collect a more representative sample of the population.
